# Structural insights of the enzymes from the chitin utilization locus of *Flavobacterium johnsoniae*

**DOI:** 10.1038/s41598-020-70749-w

**Published:** 2020-08-13

**Authors:** Scott Mazurkewich, Ronny Helland, Alasdair Mackenzie, Vincent G. H. Eijsink, Phillip B. Pope, Gisela Brändén, Johan Larsbrink

**Affiliations:** 1 Wallenberg Wood Science Center, Department of Biology and Biological Engineering, Chalmers University of Technology, 412 96 Gothenburg, Sweden; 2 Department of Chemistry, Faculty of Science and Technology, UiT, The Arctic University of Norway, 9037 Tromsø, Norway; 3 Faculty of Chemistry, Biotechnology and Food Science, Norwegian University of Life Sciences (NMBU), 1432 Ås, Norway; 4 Department of Animal and Aquacultural Sciences, Faculty of Biosciences, Norwegian University of Life Sciences (NMBU), 1432 Ås, Norway; 5 Department of Chemistry and Molecular Biology, University of Gothenburg, 405 30 Gothenburg, Sweden

**Keywords:** SAXS, X-ray crystallography

## Abstract

Chitin is one of the most abundant renewable organic materials found on earth. The chitin utilization locus in *Flavobacterium johnsoniae*, which encodes necessary proteins for complete enzymatic depolymerization of crystalline chitin, has recently been characterized but no detailed structural information on the enzymes was provided. Here we present protein structures of the *F. johnsoniae* chitobiase (*Fj*GH20) and chitinase B (*Fj*ChiB). *Fj*GH20 is a multi-domain enzyme with a helical domain not before observed in other chitobiases and a domain organization reminiscent of GH84 (β-*N*-acetylglucosaminidase) family members. The structure of *Fj*ChiB reveals that the protein lacks loops and regions associated with *exo*-acting activity in other chitinases and instead has a more solvent accessible substrate binding cleft, which is consistent with its *endo*-chitinase activity. Additionally, small angle X-ray scattering data were collected for the internal 70 kDa region that connects the N- and C-terminal chitinase domains of the unique 158 kDa multi-domain chitinase A (*Fj*ChiA). The resulting model of the molecular envelope supports bioinformatic predictions of the region comprising six domains, each with similarities to either Fn3-like or Ig-like domains. Taken together, the results provide insights into chitin utilization by *F. johnsoniae* and reveal structural diversity in bacterial chitin metabolism.

## Introduction

Chitin is as a key component of arthropod exoskeletons and fungal cell walls, and as such one of the most abundant renewable organic materials found on earth. The long chitin polysaccharides, consisting of β(1 → 4)-linked *N*-acetyl-d-glucosamine (GlcNAc) units, share many properties with cellulose; both polysaccharides are completely insoluble and coalesce into crystalline fibers, and their decomposition is highly challenging. Metabolism of chitin and cellulose proceeds through similar systems, where multiple enzymatic activities are required. *Endo*-acting enzymes cleave amorphous regions of the crystals, while *exo*-acting processive enzymes depolymerize the chains from either the reducing- or non-reducing ends. In addition, lytic polysaccharide monooxygenases (LPMOs) introduce chain breaks in crystalline regions, whereas β-glycosidases complete the hydrolysis by converting oligosaccharides into monosaccharides^1,2^. The chitinolytic machinery of the bacterium *Serratia marcescens* has served as a model system for enzymatic chitin turnover, and consists of five core enzymes: *Sm*ChiA and B (*exo*-acting processive chitinases), *Sm*ChiC (*endo*-acting non-processive chitinase), CBP21 (LPMO), and a chitobiase converting chitooligosaccharides (CHOs) into GlcNAc^2^.

Bacteria belonging to the Bacteroidetes phylum are well-known as efficient metabolizers of complex polysaccharides, and many of these species contain so-called polysaccharide utilization loci (PULs) which target distinct glycans^3^. PULs are discrete gene clusters, comprising all of the proteins needed to bind, transport and enzymatically deconstruct complex polysaccharides, and many PULs targeting both plant- and microbial glycans have been characterized in recent years^4–6^. No culturable Bacteroidete has yet conclusively been shown to deconstruct cellulose via the actions of a PUL^7^, but we recently identified and characterized a chitin utilization locus (ChiUL) from the aerobic soil bacterium *Flavobacterium johnsoniae*^8^. An atypical feature of the ChiUL compared to other characterized PULs from gut bacteria is that the main chitinase, *Fj*ChiA, is not attached to the outer membrane but is instead solubly secreted into the extracellular milieu by the Type IX secretion system^8–10^. This possibly reflects the fact that the environment of *F. johnsoniae* is less nutrient dense compared to the gastrointestinal tracts of animals and may require both protein secretion and cellular motility for efficient scavenging for resources^11^.

The *F. johnsoniae* ChiUL consists of 11 genes: two SusC/D-like pairs for oligosaccharide capture and transport, an inner membrane transporter, a two-component sensor/regulator pair, and four enzymes. While one of the enzymes of the locus, a predicted glucosamine-6-phosphate deaminase, proved impossible to produce by heterologous expression, the three remaining enzymes directly targeting chitin or CHOs were studied in detail. The large (~ 158 kDa) *Fj*ChiA enzyme is essential for efficient chitin depolymerization and growth on chitin^8,10^, and consists of two catalytic domains, *Fj*ChiA_N (N-terminal, *exo*-acting) and *Fj*ChiA_C (C-terminal, *endo*-acting), both from glycoside hydrolase family 18 (GH18) and sharing only ~ 20 to 30% sequence identity to previously characterized family members. *Fj*ChiA_N and *Fj*ChiA_C are connected by a ~ 70 kDa segment, *Fj*ChiA_M, which lacks significant similarity in primary structure to any known proteins but was shown to bind crystalline polysaccharides and may as such present a novel carbohydrate-binding module (CBM) motif^8^. The chitin depolymerization activity of the native full-length *Fj*ChiA was dramatically better than a combination of the two terminal catalytic domains, indicating the importance for the *Fj*ChiA_M domain in this multi-domain enzyme. The remaining GH18 chitinase of the locus, *Fj*ChiB (*endo*-acting), was active on chitin and CHOs, but to a much lesser extent compared to *Fj*ChiA. The fourth enzyme, the *Fj*GH20 chitobiase/*N*-acetylglucosaminidase, was active only on CHOs.

In the carbohydrate-active enzymes database (CAZy; https://www.cazy.org^12^), GH18 is a large family comprising over 20,000 members with close to 500 having been biochemically characterized to date. Of these, 93 enzymes are currently listed as structurally determined (April 2020). While this is a small subset compared to the family size, the number of solved structures provides deep insight into the enzymes’ structure–function relationships. The catalytic domains of GH18 enzymes consist of (β/α)_8_ barrels which are often appended by CBMs or other chitin-binding structures that are important for overall enzyme efficiency^13–15^. A notable feature present in some GH18 members is a region/domain inserted between the β-strand 7 and α-helix 7 of the barrel, referred to as the chitinase insertion domain (CID), which is proposed to aid in defining the processivity of the enzymes by forming a (partial) tunnel over a bound polysaccharide chain. Structural information on GH20 enzymes is sparser, with 22 structures currently deposited to the protein data bank (PDB). While several GH20 enzymes have been shown to be multi-modular and the family as a whole contains a range of substrate specificities, the enzymes all comprise a core catalytic domain consisting of a (β/α)_8_ barrel that carries conserved catalytic residues^16–20^.

In our previous work, we solved the structures of the surface-tethered SusD-like proteins CusD_I_ and CusD_II_, however the structural basis for the activity of the enzymes of the ChiUL remained unresolved^8^. Here we present the crystal structures of the *Fj*ChiB chitinase and the *Fj*GH20 chitobiase, as well as a small-angle X-ray scattering (SAXS) envelope of the novel *Fj*ChiA_M domain. The protein structures reveal features consistent with their enzymatic activities and reveal a novel domain in *Fj*GH20. The ab initio modelling of the *Fj*ChiA_M SAXS envelope indicated an elongated protein which, supported by bioinformatic analyses, is suggested to be comprised of distinct modules. Collectively, the results add to our understanding of the structure–function relationships of these important enzymes that are involved in the metabolism of the abundant polysaccharide chitin.

## Results and discussion

### Structure of *Fj*GH20

**Figure 1:**
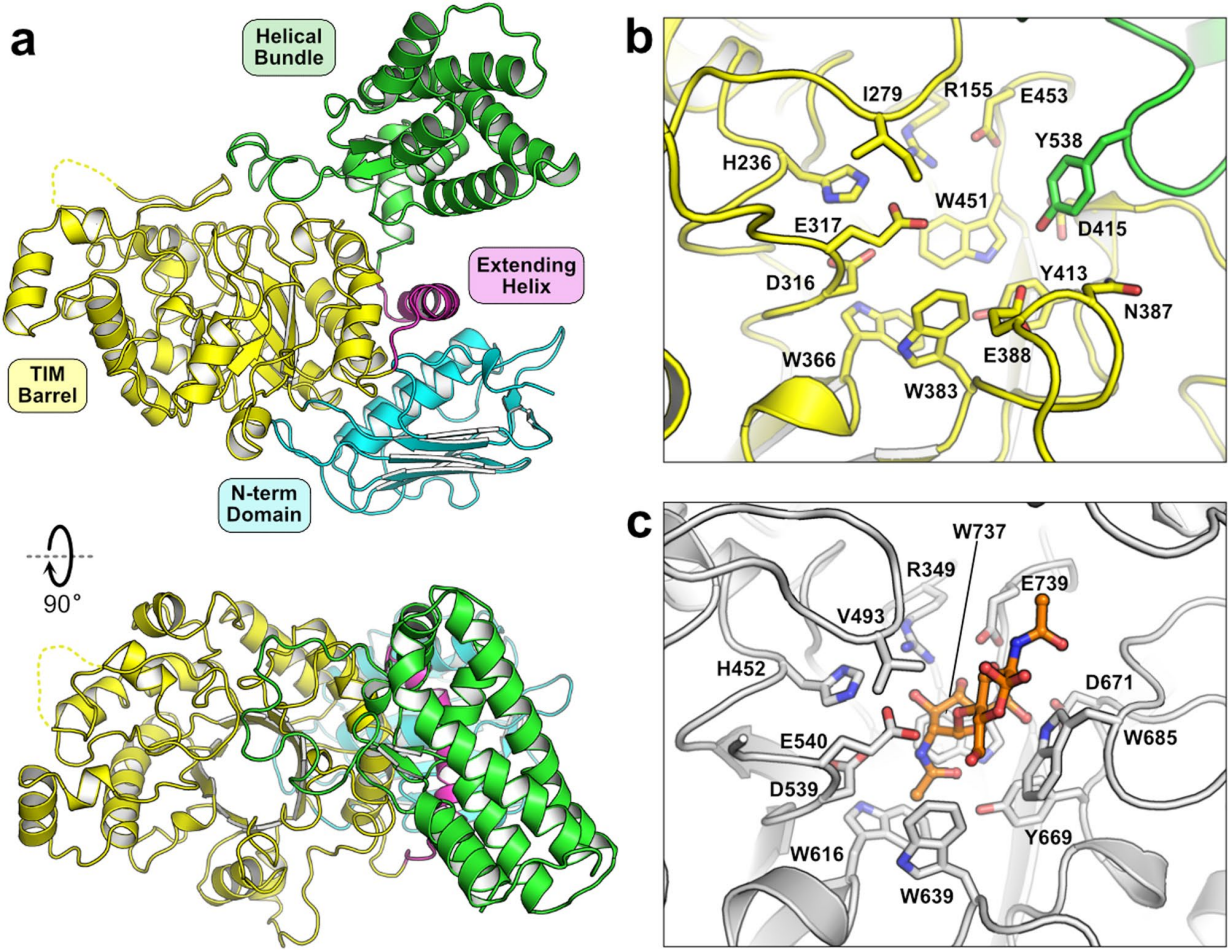
Structure of *Fj*GH20. The overall structure of the enzyme is shown with the individual domains annotated by color **(a)**. The active site architectures of *Fj*GH20 **(b)** and *Sm*GH20 (**c**; PDB accession 1qbb) are shown with key residues lining the substrate-binding pocket. The structure of *Sm*GH20^20^ was determined in complex with chitobiose (orange carbons). A notable difference between the two enzymes is the presence of a tyrosine (Tyr538) from a loop of the helical bundle domain in *Fj*GH20 filling the void left by the absence of a conserved tryptophan (Trp685 in *Sm*GH20), which is involved in substrate binding by ring stacking with a GlcNAc residue in chitobiose.

GH20 family members are multi-domain proteins with a TIM-barrel domain containing the catalytic site. The predicted catalytic domain of *Fj*GH20 shares 25 to 38% sequence identity with previously characterized GH20 family members, but the overall amino acid sequence identity is as low as 15% compared to certain studied enzymes. Based on these data, we hypothesized that *Fj*GH20 might also contain structural features or domains previously not seen in other family members. Thus, to illuminate these features we pursued structural determination of the *Fj*GH20 by X-ray crystallography and were able to solve the protein structure from diffraction data extending to 1.70 Å resolution. The crystallographic asymmetric unit contained two protein molecules whose interaction gives rise to a buried surface area of ~ 11%, as determined by PISA^21^. Analysis of the protein in solution by size exclusion chromatography was consistent with a monomer unit being the prominent species (≥ 80%) and thus the dimer observed in the crystal structure is likely not of biological relevance. Each protomer consists of three domains: a central domain (residues 145–502) composed of a (β/α)_8_ TIM-barrel, an N-terminal domain (residues 1–144) comprising a six-stranded β-sheet and two α-helices where the helices are sandwiched between the β-sheet and the central domain, and a C-terminal domain (residues 504–673) consisting of an eight-helix bundle with two short β-strands (Fig. 1). An α-helix (residues 481–503) extends from and across α-helix 8 of the TIM-barrel to connect the central domain to the C-terminal helical bundle. Most of the polypeptide chains are well defined in the electron density except for residues 243–261 and 421–429 in both protomers, which are not visible and are poorly defined, respectively, suggesting a greater degree of flexibility of these regions.

### Structural features of *Fj*GH20 and comparison to homologous structures

The *Fj*GH20 N-terminal and central domains, although only sharing up to 35% sequence identity over the domains, are closely related in structure to several GH20 members, such as the β-hexosaminidases from *Bacteroides thetaiotaomicron* (BT0459; PDB accession 6q63) and *Homo sapiens* (PDB accession 1o7a; root mean square deviation of Cα atoms ~ 2.5 Å, as determined by DALI^22^). The N-terminal domain of GH20 family members is ubiquitous and bears structural resemblance to certain CBMs, but a defined biological role for this domain remains undetermined. The catalytic site for the *N*-acetylhexosaminidase activity in these enzymes is found in the cleft of the TIM-barrel. Several of the active site residues shown to be important for substrate binding and catalysis in GH20 members are conserved in *Fj*GH20, including the H-x-G-G-D-E motif where a glutamic acid (Glu317) is proposed to act as a general acid/base in the reaction. The aspartic acid in this motif (Asp316), along with a conserved tyrosine residue (Tyr413), are proposed to position and polarize the *N*-acetyl group for the substrate-assisted catalytic mechanism^20,23,24^. The *Fj*GH20 substrate-binding pocket is distinct in one significant way compared to the archetypical GH20 from *S. marcescens* (*Sm*GH20)^20,25^ in that *Fj*GH20 lacks the extended loop that is located between β-strand 7 and α-helix 7 in *Sm*GH20 (Fig. 1). This alteration of the *Fj*GH20 TIM-barrel leads to loss of a conserved active site tryptophan residue (Trp685), which is involved in sugar binding in the + 1 subsite^26^ of *Sm*GH20. Interestingly, a portion of a loop extending from the *Fj*GH20 helical bundle domain wraps around, and closes off, one side of the active site cleft in the TIM barrel with a tyrosine residue (Tyr538) that projects its sidechain into a position similar to the Trp685 residue in *Sm*GH20. A similar arrangement occurs in the homologous human β-hexosaminidase, which also has a short loop between β-strand 7 and α-helix 7 of the TIM-barrel and has a tyrosine residue in a position similar to Tyr538 in *Fj*GH20. Interestingly, in the human protein the tyrosine residue originates from a loop in a different protomer^27^. It seems unlikely that this different feature of the substrate-binding sites significantly affects overall activity since *Fj*GH20, human β-hexosaminidase, and a GH20 enzyme from *B. thetaiotaomicron*, which lacks an analogous aromatic residue, maintain enzymatic activity toward their target substrates^8,28^.

The *Fj*GH20 C-terminal helical bundle is a distinct domain amongst structurally determined GH20 family members. The GH20 family is distantly related to GH84, which also contains enzymes with *N*‐acetyl‐β‐hexosaminidase activity and its members display a similar structural architecture, i.e. an N-terminal β-sheet domain and a (β/α)_8_ TIM-barrel catalytic domain containing GH20-like catalytic residues. Some GH84 members, such as GH84C (NagJ) from *Clostridium perfringens*^29,30^, contain a C-terminal helical bundle analogous to that observed in *Fj*GH20 (Supplemental Fig. 1). As in *Fj*GH20, a loop from the helical bundle in NagJ also lines one face of the substrate binding pocket resulting in a tyrosine side chain being positioned analogously to Tyr538 in *Fj*GH20 and Trp685 in *Sm*GH20. In NagJ, this helical domain appears to act as a bridging domain between the catalytic TIM-barrel and multiple additional domains found closer to the C-terminus. While only distantly related in sequence (8% sequence identity shared between the full-length *Fj*GH20 and the homologous domains of NagJ), the presence of an analogous C-terminal domain in *Fj*GH20 to GH84 enzymes may suggest a closer relationship between the two GH families than has been previously suggested and/or could be a remnant of their common ancestry.

### Structure of *Fj*ChiB

**Figure 2:**
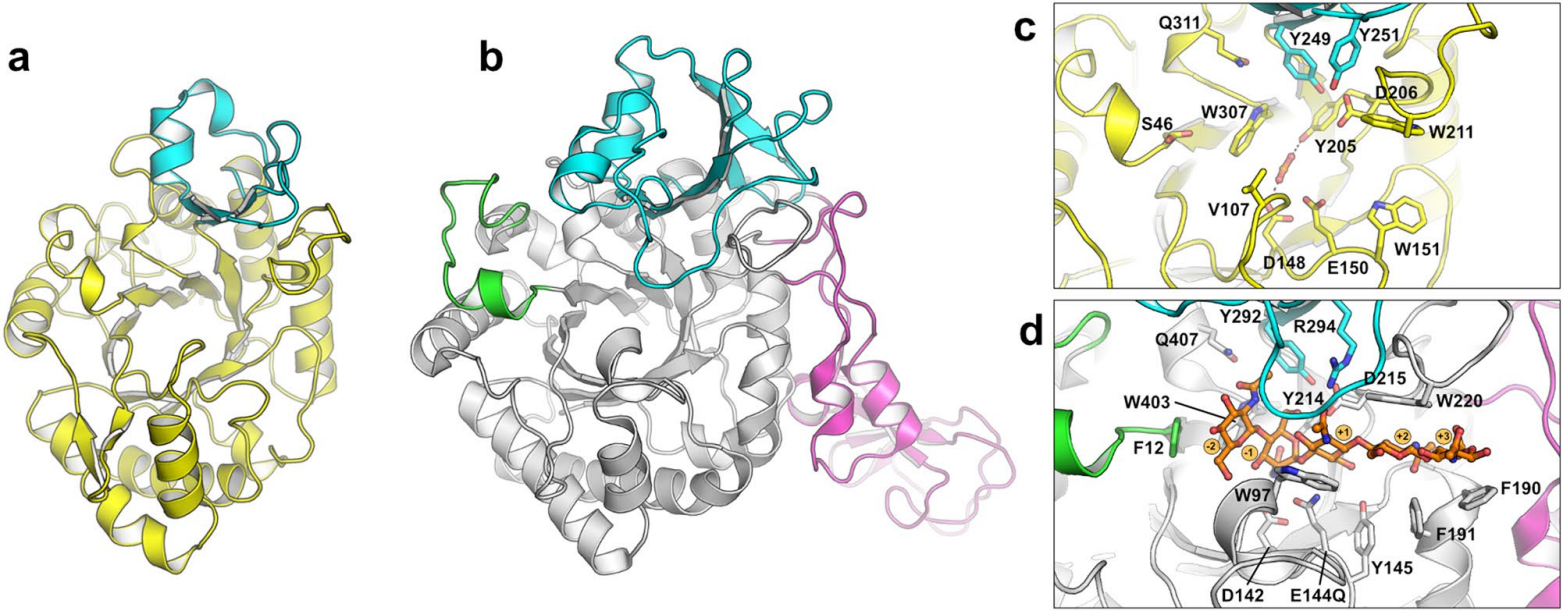
Structure of *Fj*ChiB. The overall structure of *Fj*ChiB **(a)** is shown next to *Sm*ChiB (**b**; PDB accession 1e6n). The chitinase insertion domain (CID) of each protein is colored cyan, and the -3-site capping loop and CBM family 5 domain of *Sm*ChiB are colored green and magenta, respectively. The active site architectures of *Fj*ChiB **(c)** and *Sm*ChiB **(d)** show the key residues lining the substrate binding clefts. The structure of *Sm*ChiB contained the catalytic residue substitution E144Q enabling the determination in complex with chitopentaose (orange sticks). Notably, the smaller CID and the lack of motifs equivalent to the capping loop of *Sm*ChiB lead to a more exposed substrate binding cleft in *Fj*ChiB.

Although many GH18 chitinase structures have been determined to date (> 75 from distinct species), ChiB only shares up to 30% sequence identity to enzymes with solved structures and its structure could therefore provide novel insights into the GH18 family as a whole. To investigate these features, we pursued structural determination by X-ray crystallography and were able to solve the structure of *Fj*ChiB to 1.63 Å resolution. The asymmetric unit contained one protein molecule without contacts indicative of oligomerization. The overall *Fj*ChiB structure is similar to other structurally determined GH18 chitinases, having a (β/α)_8_ TIM-barrel fold and containing a CID between strand 7 and helix 7 of the (β/α)_8_-barrel (residues 249–286; Fig. 2). The electron density is well defined with only 13 residues from the N-terminus not possible to resolve in the final model. Notably, electron density for three residues of the C-terminal histidine tag used for affinity chromatography purification was resolved and modelled close to a symmetry related molecule’s substrate binding cleft.

### Structural features of *Fj*ChiB and comparison to homologous structures

The catalytic motif (DxxDxDxE) common amongst GH18 family members, which supports a substrate-assisted catalytic mechanism^2^, is conserved in *Fj*ChiB (Asp146-Val147-Asp148-Leu149-Glu150; Fig. 2). Electron density consistent with a formate molecule, likely from the crystallization solution, was found in the active site positioned by hydrogen bonds with the hydroxyl moiety of Tyr205 and the carboxyl moiety of Glu150. The orientation and position of the formate molecule is similar to that of the acetyl group of a GlcNAc unit bound in the -1 subsite in several GH18 ligand complex structures. The overall architecture of the substrate-binding cleft of *Fj*ChiB is similar to that of other GH18 chitinases. Relative to *Sm*ChiB, a processive *exo*-acting enzyme that is amongst the best studied GH18 enzymes, the binding cleft of *Fj*ChiB has two distinct differences, described in more detail below, which leads to the cleft being more open and exposed to the bulk solvent.

In *Sm*ChiB, a small insertion between β-strand 1 and α-helix 1 leads to a capping of the cleft at the -3 site ^14^, which likely explains why this enzyme favors *exo*-binding (at the non-reducing end of a chitin chain), rather than *endo*-binding^2,31^. *Fj*ChiB lacks this insertion and instead shows more similarity to the GH18 ChtII from the insect pest *Ostrinia furnacalis*, which also lacks this insertion and has been shown to be able to bind longer oligosaccharides beyond the -3 site^32^. Like several GH18 chitinases, *Fj*ChiB has a CID inserted between strand 7 and helix 7 of the (β/α)_8_-barrel which folds into a distinct domain and builds up one face of the active site cleft. In *Sm*ChiB, this region is large and folds over one end of the cleft effectively forming a tunnel and shielding the − 1 and + 1 sites from the bulk solvent^2,14^. There is significant diversity amongst GH18 chitinase members in the CID region, both in length and sequence, and the CID in *Fj*ChiB, while similar in overall structure, is shorter than the one found in *Sm*ChiB, leading to a much more open cleft at the + 1 and + 2 sites (Fig. 2). A small or absent CID, leading to a more exposed binding cleft, has been observed in other GH18 chitinases, such as the *O. furnacalis* ChtII^32^, and is commonly associated with *endo*-acting activity. Previous work has shown *Fj*ChiB to be an *endo*-acting chitinase^8^ and the openness of the active site cleft is consistent with this activity.

### Structural investigation of the multi-modular *Fj*ChiA

*Fj*ChiA is indispensable for the growth of *F. johnsoniae* on crystalline chitin. Between its two GH18 chitinase domains, the protein contains a middle domain (*Fj*ChiA_M) with carbohydrate-binding functionality, which lacks close similarity to any previously studied proteins^8^. Attempts to crystallize either the full-length protein or the middle domain of *Fj*ChiA were unsuccessful. However, thanks to high sequence similarity, reliable homology modeling of the *Fj*ChiA N- and C-terminal GH18 chitinase domains was possible using PHYRE2^33^. The modelling resulted in high-confidence structure predictions of TIM-barrel proteins consistent with both *Fj*ChiA_N and *Fj*ChiA_C belonging to the GH18 family (Supplemental Fig. 2). Both domains contain the conserved DxxDxDxE catalytic motif. Of characterized chitinases *Fj*ChiA_N is most similar to ChiW from *Paenibacillus* sp. str. FPU-7, a chitinase from *Bacillus circulans* WL-12, and *Sm*ChiA, while *Fj*ChiA_C is most similar to chitinases from *Bacillus cereus* NCTU2, *Chromobacterium violaceum*, and *Sm*ChiC (Supplemental Fig. 3). Our previous functional characterization of the individual catalytic domains suggested that *Fj*ChiA_N and *Fj*ChiA_C were *exo*- and *endo*-acting chitinases, respectively^8^. As discussed above, in GH18 members the presence of a large CID, that partially covers the substrate-binding cleft, is associated with a higher degree of *exo*- and processive characteristics while a smaller or absent CID, leading to a more open binding cleft, is associated with *endo*-acting activities^2^. As illustrated in Supplemental Figs. 2 and 3, a large and extensive CID domain is present *Fj*ChiA_N whereas the domain is much smaller in *Fj*ChiA_C, consistent with the observed enzyme activities^8^.

**Table 1:** SAXS data collection and analysis parameters.

	*Fj*ChiA_M
**Data collection**	
Date	May 5, 2019
Source	BL4-2 at SSRL
Wavelength (Å)	1.127
Sample to detector distance (m)	1.8025
Exposure time per frame (s)	1.0
Protein concentration (mg/mL)	5.0
**Data analysis**	
*R*_*g*_ (nm) [from Guinier approximation]	4.72
*R*_*g*_ (nm) [from *P(r)* function]	4.75
*D*_max_ (nm) [from *P(r)* function]	16.3
*V*_porod_ (nm^3^)	82.7
SASBDB accession	SASDHU9

**Figure 3:**
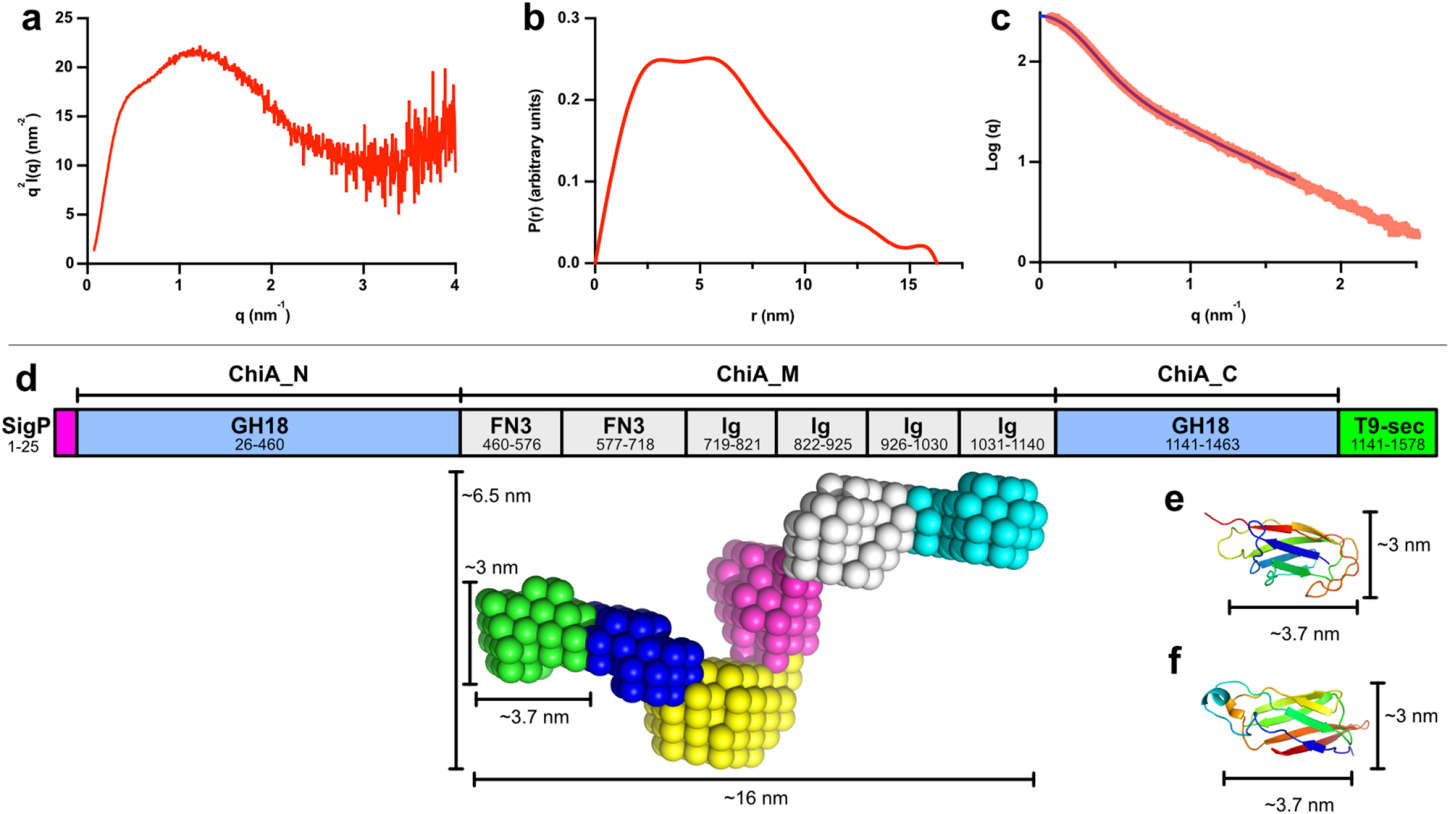
Small angle X-ray scattering of *Fj*ChiA_M. The Kratky plot **(a)** indicates a structured protein with some degree of flexibility while the pair distance distribution function **(b)** is consistent with an elongated and modular protein. **(c)** The fit of the ab initio envelope is shown as a blue line (χ^2^ = 1.20; generated in ATSAS as described in the methods) alongside the experimental data shown in red. **(d)** The domain organization of *Fj*ChiA, including regions predicted for the signal peptide (SigP) and type-9 secretion signal (T9-sec), shown along with the *Fj*ChiA_M ab initio SAXS envelope with putatively defined discrete modules uniquely colored. The Fn3-like **(e)** and Ig **(f)** domains from the pilin protein BcpA from *Bacillus cereus* (PDB accession 3kpt) and an antifreeze protein from *Marinomonas primoryensis* (PDB accession 4p99), respectively, display between 20–30% sequence identity with the putatively annotated regions in *Fj*ChiA_M.

In the absence of atomic-level structural information, and to gain better insights into the overall structure of the multi-modular *Fj*ChiA, we utilized small angle X-ray scattering (SAXS) to determine a solution structure of *Fj*ChiA_M. Unfortunately, the full-length protein suffered from both aggregation and radiation damage issues, even when utilizing SAXS measurements coupled to size exclusion chromatography, and the data could not be utilized for analysis. The *Fj*ChiA_M protein, however, proved to be much more amenable to the technique and allowed for the generation of a low-resolution model of the domain (Table 1). Analysis of the data by a Kratky plot and the pair distance distribution function, P(*r*), indicated that the protein is modular and elongated with some degree of flexibility^34^ (Fig. 3). The ab initio calculation of the SAXS molecular envelopes of *Fj*ChiA_M consistently yielded an elongated protein comprised of 6 distinct modules each between 30 to 40 Å in length and ~ 30 Å wide (Fig. 3). The envelope of *Fj*ChiA_M is slightly compressed with a small rotation between the third and fourth modules.

The *Fj*ChiA_M domain lacks significant sequence similarity to any previously characterized or structurally determined proteins, as determined by NCBI BLAST. However, protein structure predictions using PHYRE2^33^ suggest that *Fj*ChiA_M is composed of six modules comprised of two Fn3-like domains (residues 471–577 and 578–718) followed by four immunoglobulin (Ig)-like domains (residues 719–821, 822–925, 926–1,030, and 1,031–1,140). Fn3-like and Ig-like domains are similar to each other, with both comprising seven to nine strands arranged into two β-sheets that pack onto each other^35^. However, Fn3-like domains tend to have shorter strands and longer intervening loop regions compared to Ig-like domains. The two *Fj*ChiA_M Fn3-like domains share 36% identity to each other and are both most closely related (20% and 22% sequence identity, respectively) to the Fn3-like domain of the pilin protein BcpA from *Bacillus cereus* (PDB accession 3kpt). The first two Ig-like domains share 75% sequence identity while the last two shares only 20 to 30% identity with each other and with the first two. Structure predictions of these last four domains are consistent with each comprising an Ig-like domain with each sharing between 20 to 30% sequence identity to Ig-like domains from the antifreeze protein (*Mp*AFP) from *Marinomonas primoryensis*^35^. *Mp*AFP is a large (1.5 MDa) protein comprised of > 100 tandem Ig-like domains that are proposed to extend and project the ice-binding domain of the protein away from the cell. Both Fn3-like and Ig-like domains are commonly found in extracellular carbohydrate-active enzymes and, while sometimes displaying weak carbohydrate-binding ability, it has been suggested that they can play a role in loosening and exfoliating chains from fibrous polysaccharides^36^. In *Sm*ChiA, an Fn3-like domain is connected to the catalytic domain in close proximity to the active site and may have a role in the interaction with polysaccharide substrates^37,38^, and it is possible that Ig-like modules could also have substrate interaction roles.

**Figure 4:**
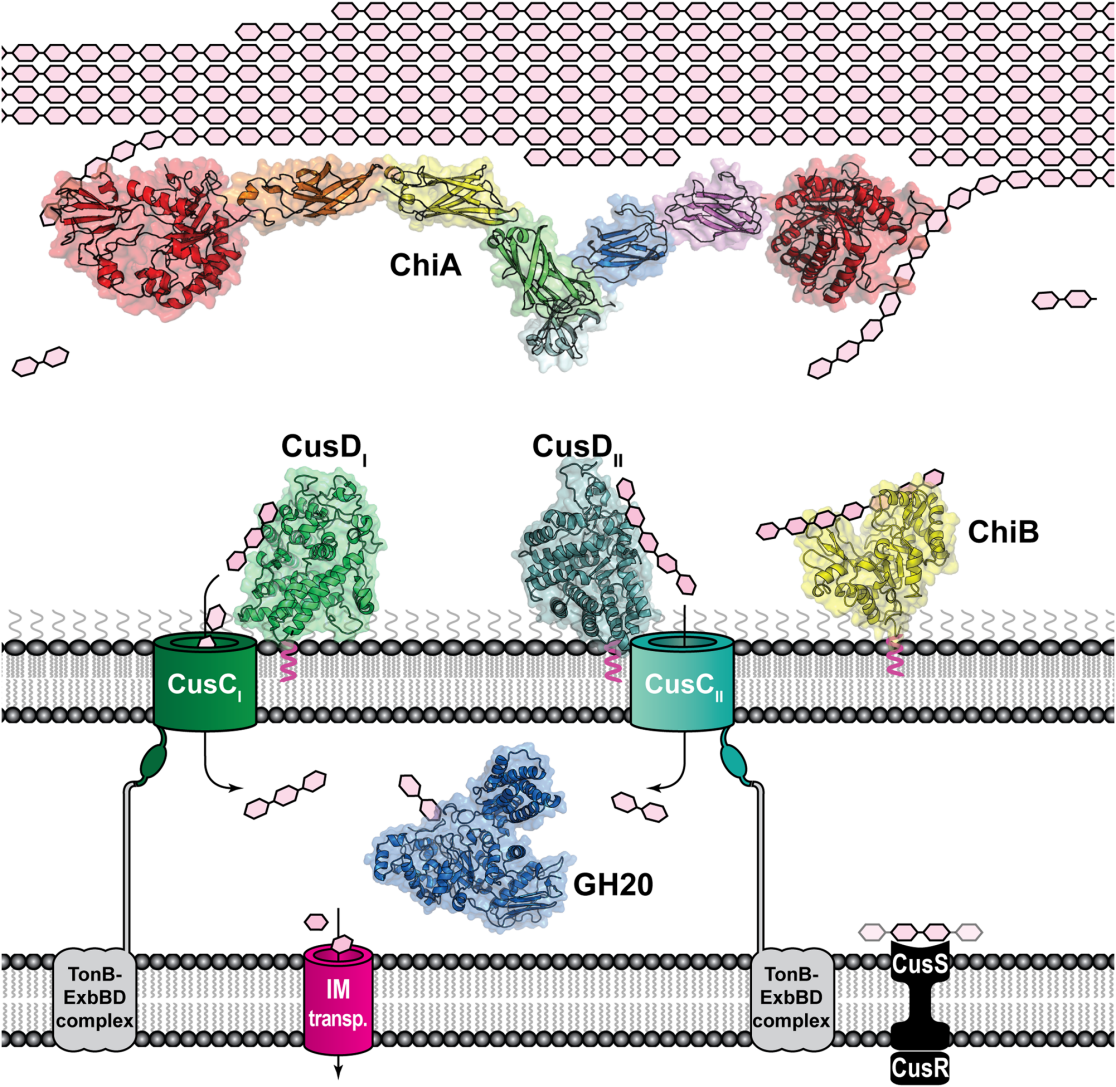
Proposed model of the ChiUL. *Fj*CusD_I_ (PDB accession 5j90), *Fj*CusD_II_ (PDB accession 5j5u), and *Fj*ChiB are outer membrane-bound lipoproteins where the former two bind oligosaccharides and facilitate import into the periplasm and the latter is an *endo*-acting chitinase. *Fj*GH20 is a periplasmic chitobiase that cleaves imported oligosaccharides into GlcNAc for further metabolism. A modelled structure of ChiA based on homology models of the terminal GH18 chitinases, *Fj*ChiA_N and *Fj*ChiA_C, and spanned by homology models of the Fn3- and Ig-like domains of *Fj*ChiA_M fitted into the modelled SAXS envelope is visualized bound to, and possibly exfoliating, polysaccharides from insoluble chitin crystals.

Collectively, the solution scattering results support the bioinformatic prediction that the *Fj*ChiA_M domain is composed of distinct modules which likely fold similarly to Fn3-like and Ig-like domains. Further, the protein is observed as elongated in solution in a fashion similar to “beads on a string” where each bead may sit on crystalline chitin and exfoliate chains for the two terminal chitinase domains. Our previous biochemical characterization^8^ showed that the *Fj*ChiA_M domain adheres to several insoluble and crystalline polysaccharides including α- and β-chitin and cellulose. The elongated conformation of the protein may be a feature that is important not only for adhesion/exfoliation but also for tethering *Fj*ChiA_N and *Fj*ChiA_C together physically for increased cooperativity between the domains. A tentative model of the *Fj*ChiA solution structure together with the entire ChiUL machinery is presented in Fig. 4.

## Conclusions

Together with our previous characterization, the data presented here represent a holistic structural view of the chitin-interacting proteins of the *F. johnsoniae* ChiUL, including both carbohydrate-binding proteins and enzymes (Fig. 4). Our results in addition provide new structural information for both the GH18 and GH20 families. The *Fj*GH20 structure reveals novel features previously not seen in structures of GH20 members, and the results suggest a stronger connection between families GH20 and GH84. *Fj*ChiA is an exceptionally powerful multi-modular chitinolytic enzyme and our SAXS model of its internal domain, *Fj*ChiA_M, showcases how *F. johnsoniae* has evolved this multidomain chitinase to form a complex enzyme with the terminal chitinase domains separated by an extended protein ‘spacer’ that also has carbohydrate-binding abilities. The exact mechanism of substrate-binding for the Fn3- and Ig-like domains of *Fj*ChiA_M remains elusive, but the current model provides a useful template for future studies. Further insights into the structure, dynamics and functional abilities of multi-catalytic molecular “machines” such as *Fj*ChiA may have implications not only for understanding chitin deconstruction, but also for understanding, and eventually designing and optimizing, enzymatic deconstruction of other recalcitrant polysaccharides.

## Methods

### Structure determination of *Fj*GH20

The protein was produced and purified as previously reported^8^. Size exclusion chromatography for oligomerization analysis was performed on a HiLoad Superdex 200 16/60 column and an ÄKTA Explorer (GE Healthcare) using an isocratic gradient of 50 mM Tris pH 8.0 with 250 mM NaCl. Molecular weights were determined by standard curve using the Gel Filtration Markers Kit for Protein Molecular Weights 12–200 kDa (Sigma) as standards. Initial crystallization conditions were screened for using a Phoenix crystallization robot (Art Robbins Instruments) by the sitting-drop vapor-diffusion method. About 400 in-house made conditions were screened by using MRC plates with a 60 µl reservoir solution per well, and drop solutions were prepared by mixing 0.25 µl well solution and 0.25 µl protein solution at 20 mg/ml. Further optimization in Hampton 24-well hanging drop plates using 500 µl reservoirs and 1 µl + 1 µl drops, yielded crystals from 14% PEGMME 5 K and 0.1 M Na-Malonate pH 7.5. Diffraction data was collected at BL14.1 at Bessy (2016–04-16), and was integrated and scaled using XDS^39^ and AIMLESS^40,41^, respectively. The structure was solved by molecular replacement with Auto-Rickshaw^42,43^, with MoRDa^44^ identifying and using PDB accession 1now, human lysosomal beta-hexosaminidase^45^, as the template. An initial model was built using autobuilding in ARP/wARP^46–49^. Inspection of electron density maps was done in Coot^50^ with positional refinement in REFMAC^51^. The data collection, processing, and refinement statistics for all of the datasets can be found in Table 2.

**Table 2:** Summary of crystallographic statistics.

	*Fj*GH20	*Fj*ChiB
**Data collection**		
Date	April 14, 2016	May 3, 2017
Source	BL 14.1 at Bessy	ID30A-3 at ESRF
Wavelength (Å)	0.918409	0.9677
Space group	*P* 2_1_ 2_1_ 2_1_	*P* 1 2_1_ 1
Cell dimensions		
*a, b, c* (Å)	75.99, 124.55, 151.59	43.91, 66.44, 53.44
α, β, γ (°)	90, 90, 90	90, 98.31, 90
No. of measured reflections	1,033,044 (35,652)	163,627 (14,163)
No. of independent reflections	158,136 (7,637)	34,484 (2,962)
Resolution (Å)	49.1–1.7 (1.73–1.70)	36.4–1.6 (1.69–1.63)
*R*_merge_ (%)	7.10 (39.4)	4.30 (20.7)
CC_1/2_ (%)	99.9 (86.8)	99.8 (98.0)
Mean I/σI	17.2 (3.2)	19.7 (6.3)
Completeness (%)	99.9 (98.4)	90.7 (77.9)
Redundancy	6.5 (4.7)	4.8 (4.7)
**Refinement**		
*R*_work_/*R*_free_	0.142/0.175	0.157/0.189
No. atoms		
Protein	10,214	2,523
Ligand/ions	22	15
Water	1544	338
B-factors		
Protein	14.7	23.2
Water	23.9	34.2
RMSD		
Bond length (Å)	0.020	0.006
Bond angles (°)	1.95	0.77
PDB accession	6YHH	6XYZ

### Structure determination of *Fj*ChiB

*Fj*ChiB was produced and purified as previously reported^8^ and stored in 50 mM Tris buffer at pH 8.0 containing 50 mM NaCl. Crystallization conditions were screened for with a Mosquito robot (SPT Labtech) using the JCSG + screening kit (Molecular Dimensions) in MRC sitting drop plates. A condition which yielded a crystal hit was optimized in sitting drop plates with a reservoir volume of 40 µl and protein mixed with reservoir solution in a 1:1 ratio in 0.6 µl drop sizes using a protein stock solution at 20 mg/mL. The optimized formulation contained 0.15 M magnesium formate with 15% PEG3550 and yielded cuboid crystals within a week. An initial dataset diffracting to 1.85 Å was collected at the ESRF id23-2 (2014–12-02) which was integrated and scaled using XDS^39^ and AIMLESS^40,41^, after which the structure was determined by molecular replacement with Auto-Rickshaw^42,43^, with MoRDa^44^ identifying and using PDB accession 3fnd, a putative chitinase from *Bacteroides thetaiotaomicron*, as the search template. An initial model was built using autobuilding in ARP/wARP^46–49^. A subsequent data set diffracting to 1.63 Å was collected at the ESRF id30a3 (2017-05-03), processed with XDS^39^, and the solution defined by rigid body refinement using Phenix Refine^52^ and the previously determined *Fj*ChiB structure. Since the new data set provided an improvement in resolution, only this dataset was pursued for further refinement and deposition. Coot^50^ and Phenix Refine^52^ were used in iterative cycles of manual and computational refinement. The data collection, processing, and refinement statistics for all of the datasets can be found in Table 2.

### Small-angle X-ray scattering (SAXS) of *Fj*ChiA_M

The protein was produced and purified as previously reported^8^. X-ray scattering data were obtained at BL4-2 at the Stanford Synchrotron Radiation Lightsource (SSRL) with a Pilatus3 X 1 M detector (Dectris) operated at 11.0 keV. Full-length ChiA and ChiA_M were buffer exchanged into 50 mM Tris pH 8.0 with 250 mM NaCl and 250 μM DTT, using a HiPrep 26/10 Desalting column and an ÄKTA Explorer (GE Healthcare). The proteins were concentrated to 20 mg/mL by ultrafiltration using a Vivaspin (GE Healthcare) 10 kDa molecular weight cut-off polyethersulfone spin column and a protein dilution series was created using the ultrafiltration filtrate. 10 images with 1 s exposure, taken at a distance of 1802.5 mm, were averaged and, after background subtraction of the buffer, were utilized for analysis in the ATSAS suite version 3.0^53^. PRIMUS^54^ and GNOM^55^ were utilized to assess the data. The data for the full-length ChiA showed significant indications of aggregation and radiation damage and were not pursued further. Images of ChiA_M at concentrations of 1.25, 2.5, and 5.0 mg/mL yielded Guinier R_g_ estimates within 3% of each other with no indication of radiation damage and the data collected at 5 mg/mL were chosen for further analysis. DAMMIF^56^ was used to generate 100 models that were subsequently clustered by DAMCLUST^57^ and the top cluster was averaged by DAMAVER^58^ and then refined by DAMMIN^59^. The SAXS data collection and analysis parameters can be found in Table 1.

## Supplementary information

Supplementary Information.

## Abbreviations

PUL: Polysaccharide utilization locus
ChiUL: Chitin utilization locus
LPMO: Lytic polysaccharide monooxygenase
Sus: Starch utilization system
T9SS: Type IX secretion system
TCS: Two-component sensor/regulator system
Cus: Chitin utilization system
GH: Glycoside hydrolase family
CHO: Chitooligosaccharide
ITC: Isothermal titration calorimetry
GlcNAc: *N*-Acetylglucosamine
PDB: Protein data bank
RMSD: Root mean square deviation
CBM: Carbohydrate binding module
SAXS: Small angle X-ray scattering
