# Supplementary material for: Structural insights of the enzymes from the chitin utilization locus of Flavobacterium johnsoniae
Source: Sci Rep. 2020 Aug 13;10:13775. doi: 10.1038/s41598-020-70749-w (PMC7426924; doi:10.1038/s41598-020-70749-w)
Supplement: Supplementary file 1 — Supplementary Information. [file 41598_2020_70749_MOESM1_ESM.pdf]

Structural insights of the enzymes from the Chitin Utilization Locus of *Flavobacterium johnsoniae*

Scott Mazurkewich[a], Ronny Helland[b], Alasdair Mackenzie[c], Vincent G. H. Eijsink[c], Phillip B. Pope[c,d], Gisela Brändén[e], Johan Larsbrink[a*]

a) Wallenberg Wood Science Center, Department of Biology and Biological Engineering, Chalmers University of Technology, SE-412 96 Gothenburg, Sweden
b) Department of Chemistry, Faculty of Science and Technology, UiT - The Arctic University of Norway, NO-9037 Tromsø, Norway
c) Faculty of Chemistry, Biotechnology and Food Science, Norwegian University of Life Sciences (NMBU), NO-1432 Ås, Norway.
d) Faculty of Biosciences, Biotechnology and Food Science, Norwegian University of Life Sciences (NMBU), NO-1432 Ås, Norway.
e) Department of Chemistry and Molecular Biology, University of Gothenburg, SE-405 30 Gothenburg, Sweden

* Corresponding author: Johan Larsbrink, johan.larsbrink@chalmers.se, +46(0)317723839

Supplementary figures S1-S3

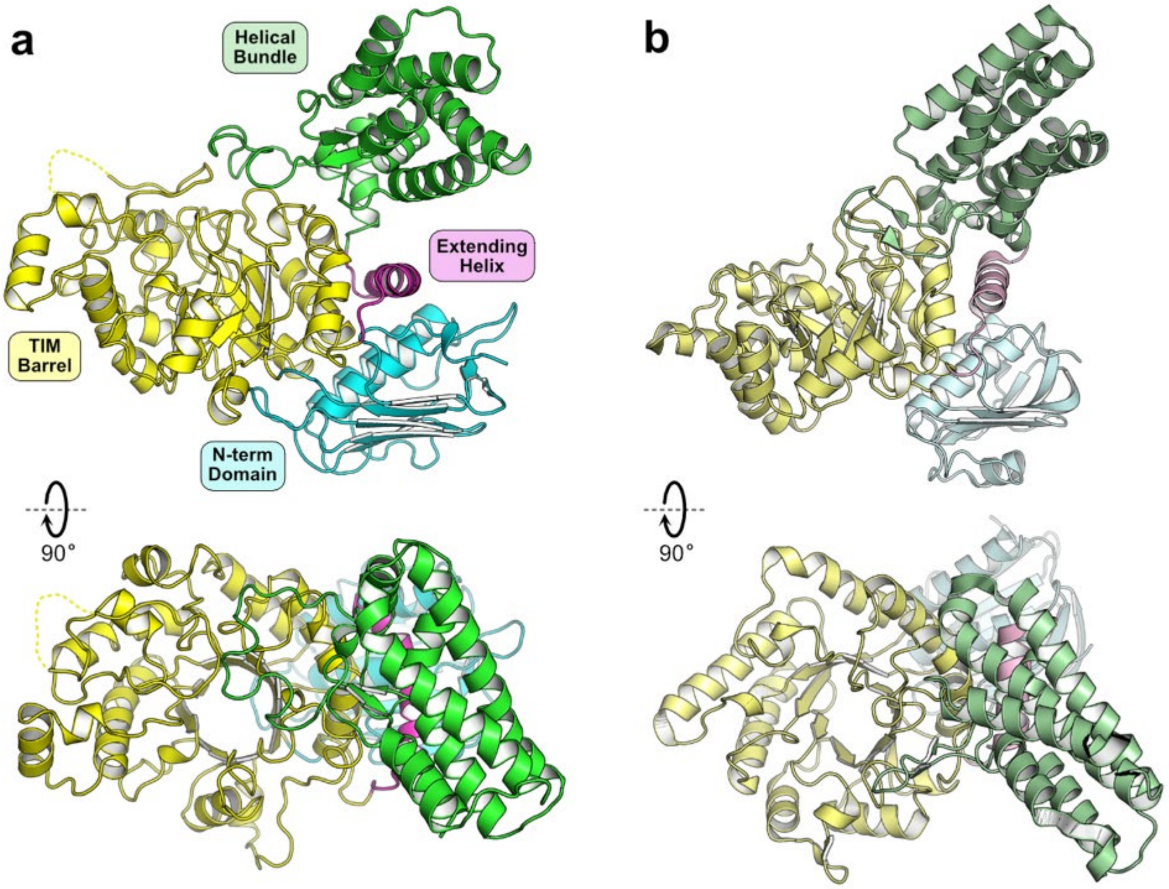

Supplemental Figure 1. Comparison of *Fj*GH20 with the GH84 member NagJ from *Clostridium perfringens*. The structures of *Fj*GH20 (a) and NagJ (b) are coloured as in Figure 1 except NagJ which is shown with pale colors.

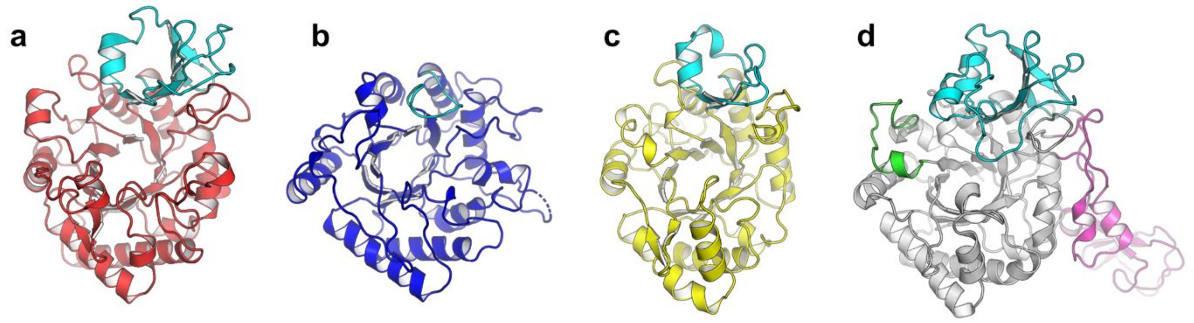

Supplemental Figure 2. Homology models of the *Fj*ChiA GH18 domains. The overall structures of the predicted *Fj*ChiA_N (a) and *Fj*ChiA_C (c) domains shown alongside the structurally determined *Fj*ChiB (c) and the *Serratia marcescens Sm*ChiB (d; PDB accession 1e6n). The chitinase insertion domains (CIDs) are colored in cyan and the *Sm*ChiB domains are colored as in Figure 2. The *Fj*ChiA_N (a) and *Fj*ChiA_C homology models were created using PHYRE2 (1) and with primary sequences of *Fj*ChiA_N and *Fj*ChiA_C mapped onto chitinases from *Paenibacillus* sp. str. FPU-7 (PDB accession 5gzt) and *Chromobacterium violaceum* (PDB accession 4tx8), respectively. Regions of the primary sequence unable to be modelled are shown as dashed lines. Note that we use a wide definition of the CID, which implies that all GH18 enzymes have a CID that varies drastically in size; previous studies have employed a narrower definition leading to the conclusion that enzymes such as *Sm*ChiC, and by analogy *Fj*ChiA_C, lack a CID.

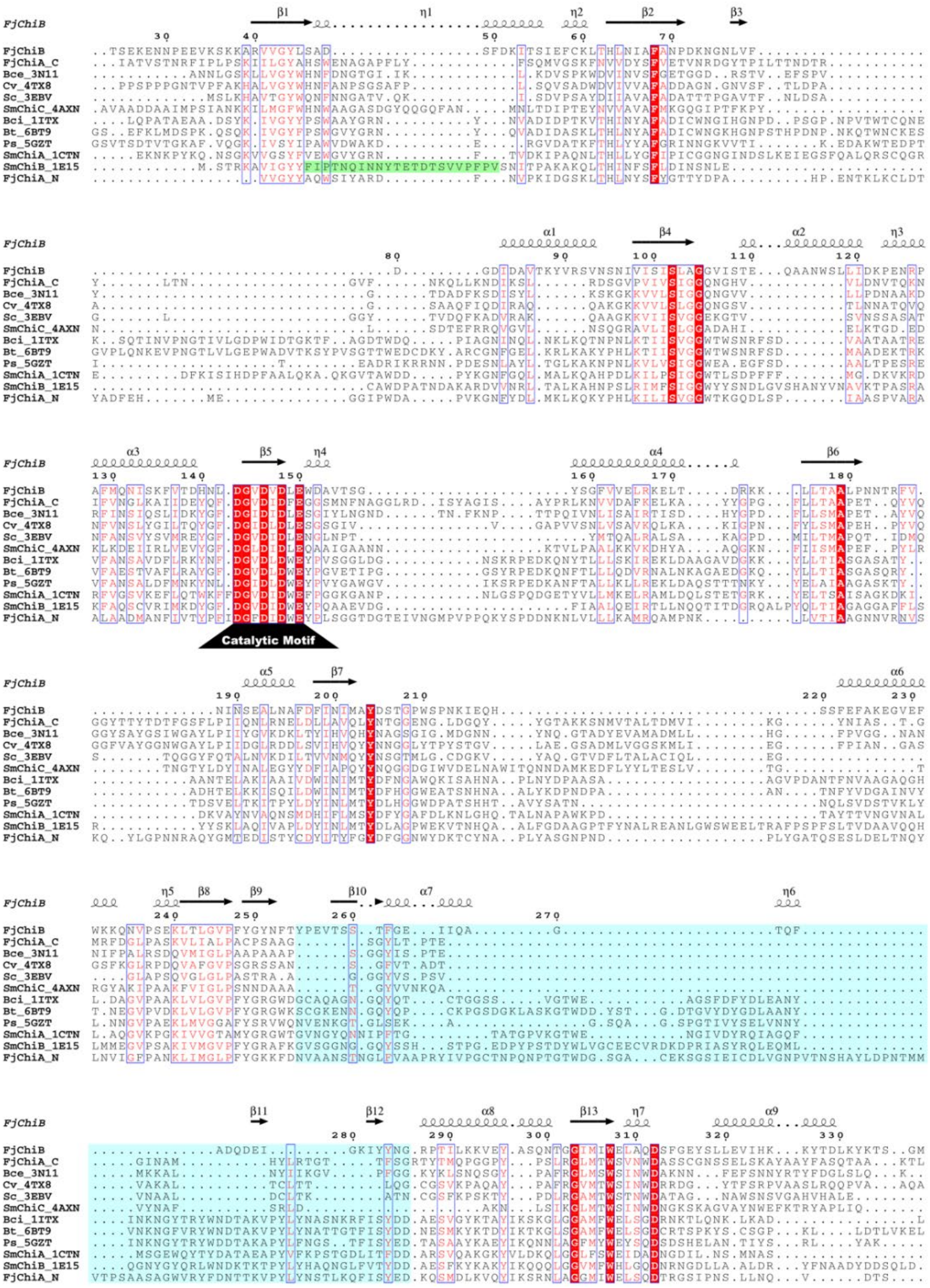

Supplemental Figure 3. Multiple sequence alignment of the catalytic domains of selected GH18 members. The chitinases from *S. marcescens* (*Sm*Chi) and *F. johnsoniae* (*Fj*Chi) are shown with the closest homologs whose protein structures are determined. Sequences are listed with their PDB accesion code and include sequences from *Bacillus cereus* NCTU2 (Bce), *C. violaceum* (Cv), *Streptomyces coelicolor* (Sc), *Bacillus circulans* WL-12 (Bci), *Bacillus thuringiensis* (Bt), and *Paenibacillus* sp. str. FPU-7 (Ps). Sequence identities between the proteins in the alignment range from 15-60% and the alignment was created with Clustal Omega (2) and visualized with Espript (3). Residue numbering above the alignment refers to *Fj*ChiB. Identical residues are shown with white text on red background while similar residues are shown with red text. The chitinase insertion domain (CID) region is highlighted in cyan, the insertion which leads to capping of the -3 site in *Sm*ChiB is highlighted in green, and the catalytic motif is annotated in black. We use the same wide definition of the CID as in Figure S2, above.

References

1. Kelley, L. A., Mezulis, S., Yates, C. M., Wass, M. N., and Sternberg, M. J. (2015) The Phyre2 web portal for protein modeling, prediction and analysis. *Nat Protoc* **10**, 845-858
2. Madeira, F., Park, Y. M., Lee, J., Buso, N., Gur, T., Madhusoodanan, N., Basutkar, P., Tivey, A. R. N., Potter, S. C., Finn, R. D., and Lopez, R. (2019) The EMBL-EBI search and sequence analysis tools APIs in 2019. *Nucleic Acids Res* **47**, W636-W641
3. Robert, X., and Gouet, P. (2014) Deciphering key features in protein structures with the new ENDscript server. *Nucleic Acids Res* **42**, W320-324
